# MCT-CNN-LSTM: A Driver Behavior Wireless Perception Method Based on an Improved Multi-Scale Domain-Adversarial Neural Network

**DOI:** 10.3390/s25072268

**Published:** 2025-04-03

**Authors:** Kaiyu Chen, Yue Diao, Yucheng Wang, Xiafeng Zhang, Yannian Zhou, Minming Gu, Bo Zhang, Bin Hu, Meng Li, Wei Li, Shaoxi Wang

**Affiliations:** 1 School of Microelectronics, Northwestern Polytechnical University, Xi’an 710072, China; kaiyu_chen@mail.nwpu.edu.cn (K.C.); diaoyue@mail.nwpu.edu.cn (Y.D.); ychwang@nwpu.edu.cn (Y.W.); zhangxiafeng@mail.nwpu.edu.cn (X.Z.); weili2019@nwpu.edu.cn (W.L.); 2 School of Information and Science, Zhejiang Sci-Tech University, Hangzhou 310018, China; guminming@zstu.edu.cn; 3 Air and Missile Defence College, Air Force Engineering University, Xi’an 710072, China; 4 Jiangsu New Vision Automotive Electronics Co., Ltd., Yangzhou 211401, China; xinzhang01@zjautomotive.com (B.Z.); 18629050073@wo.cn (B.H.); 5 Xi’an Changyuan Electronic Engineering Co., Ltd., Xi’an 710100, China

**Keywords:** driver behavior sensing, FMCW radar, MCT-CNN-LSTM, feature extraction

## Abstract

Driving behavior recognition based on Frequency-Modulated Continuous-Wave (FMCW) radar systems has become a widely adopted paradigm. Numerous methods have been developed to accurately identify driving behaviors. Recently, deep learning has gained significant attention in radar signal processing due to its ability to eliminate the need for intricate signal preprocessing and its automatic feature extraction capabilities. In this article, we present a network that incorporates multi-scale and channel-time attention modules, referred to as MCT-CNN-LSTM. Initially, a multi-channel convolutional neural network (CNN) combined with a Long Short-Term Memory Network (LSTM) is employed. This model captures both the spatial features and the temporal dependencies from the input radar signal. Subsequently, an Efficient Channel Attention (ECA) module is utilized to allocate adaptive weights to the feature channels that carry the most relevant information. In the final step, domain-adversarial training is applied to extract common features from both the source and target domains, which helps reduce the domain shift. This approach enables the accurate classification of driving behaviors by effectively bridging the gap between domains. Evaluation results show that our method reached an accuracy of 97.3% in a real measured dataset.

## 1. Introduction

Globally, road traffic accidents result in nearly 1.3 million preventable deaths and approximately 50 million injuries each year. A substantial proportion, exceeding 70%, can be linked to unpredictable driving behaviors [1,2,3]. Furthermore, road traffic accidents continue to be the primary cause of death for children and young individuals between the ages of 5 and 19, making them a significant threat to the health of children and adolescents worldwide. Thus, understanding driver behavior has the potential to substantially reduce the occurrence of these accidents. The International Data Corporation (IDC) forecasts that by 2024, the global shipments of smart vehicles featuring Advanced Driver Assistance Systems (ADASs) will reach 76.2 million units annually. As intelligent vehicle technology advances, ensuring the reliability of ADAS components such as the Brake Assistance System (BAS), Lane-Keeping System (LKS), and Adaptive Cruise Control (ACC) is crucial for drivers and other road users.

Accurately identifying driver behavior is crucial for the reliable functioning of ADASs. Researchers have explored various approaches, including vehicle-based methods (vehicle behavior) [4], physiological monitoring techniques [5], visual monitoring [6], and multi-sensor-based methods [7]. Vehicle-based approaches estimate the driver’s state by examining vehicle parameters, including acceleration and pedal force. For instance, Kanwal et al. [8] utilized vehicle accelerometer and gyroscope data collected in real traffic environments to classify three driving states: normal, slow, and aggressive driving. Zou et al. [9] employed the target vehicle’s relative distance, speed, and acceleration as feature variables, applying a Hidden Markov Model (MHMM) to categorize different driving behaviors. However, these parameters, while useful, do not directly reflect the driver’s state. Wearable sensor-based methods, including an electroencephalogram (EEG), electrocardiogram (ECG), surface electromyography (sEMG), electrooculography (EOG), and heart rate variability (HRV) detection, have also been extensively explored. Doniec et al. [10] employed EOG combined with a 1-D convolution neural network (CNN) to identify 16 types of driving behaviors, including eating and drinking, achieving an overall accuracy of 80%. Yang et al. [11] suggested an EEG-based method for driving behavior recognition, with simulated driving experiments yielding an average accuracy of 69.5% and a peak accuracy of 83.5%. However, these methods often require drivers to wear multiple electrodes that can interfere with their behavior, making them impractical for real-world applications. Among the most accurate methods to date are visual-based techniques, which monitor blinking, yawning, facial expressions, and head movements to assess the driver’s state. A recent study, [12], proposed a CLIP-based driver activity recognition system capable of detecting driver distractions (e.g., drinking, applying makeup, talking on the phone, adjusting the radio, etc.) from naturalistic driving images and videos. Nonetheless, visual systems are highly susceptible to lighting conditions, such as glare or dim environments, and can also infringe upon the driver’s privacy. Multi-sensor-based approaches to driver behavior recognition have also contributed significantly to the field. Wang et al. [13] utilized physiological signal monitoring (EEG and ECG) to detect fatigue while using in-vehicle cameras to assess behavioral deviations caused by distraction or fatigue, achieving satisfactory performance. Chen et al. [14] developed a CNN model that integrates multi-sensor data fusion to classify five types of driving behaviors, achieving an accuracy of 80%. However, multi-sensor methods require large data volumes, leading to slower processing speeds and often lack effective feature extraction. Additionally, wearable sensors can negatively affect the driver’s experience.

Frequency-Modulated Continuous-Wave (FMCW) radar, with its non-contact operation, all-weather capability, high-range accuracy, anti-interference, low latency, and miniaturization, has demonstrated significant advancements in various fields such as human gesture recognition [15], activity recognition [16], and fall detection [17]. Paper [18] introduces a gesture recognition system based on FMCW radar. The system constructs range-Doppler and range-angle maps and applies a spatiotemporal path selection algorithm to distinguish between multiple hand gestures. It then leverages dual 3D CNNs and Long Short-Term Memory Networks (LSTM) for feature extraction and classification, achieving an accuracy of 93.12% in recognizing multiple hand gestures. In paper [19], a static hand gesture recognition technique is introduced, which utilizes millimeter-wave near-field FMCW-SAR imaging. The approach captures gesture data by building a millimeter-wave near-field SAR imaging system and then applies histograms of oriented gradients (HOG) for feature extraction, Principal Component Analysis (PCA) for dimensionality reduction, and random forest for classification. As a result, this method achieves average recognition accuracies of 97% in unobstructed conditions and 93% in obstructed scenarios. Paper [20] presents a multi-target activity sensing method (CAE-MAS) based on convolutional autoencoders (CAEs) to mitigate interference in FMCW radar for multi-person activity detection. By extracting range-Doppler maps and employing the CAE network to reduce interference, this method achieves high recognition accuracy for both single-person and two-person activities at 97.13% and 73.37%, respectively, thus improving target detection performance in multi-person environments. Paper [21] introduces a method for recognizing human activities using FMCW radar signals. The method utilizes time segmentation and frequency window techniques to eliminate cross-terms, thereby generating cross-term-free time–frequency representations (TFRs) and extracting slow-time features from the radar signals, resulting in a classification accuracy of 99.51%. Paper [22] presents an unsupervised system for fall detection using FMCW radar. It involves constructing feature extractors and predictors to derive range–velocity–time features from radar signals while also learning patterns of non-fall actions. This approach demonstrates the system’s capability to generalize effectively in new scenarios. Paper [23] proposes a FMCW radar-based fall detection method. By analyzing the radar-Doppler features from human activity states and utilizing a dual-branch CNN to identify different fall directions, experimental results demonstrate that this method achieves a recognition accuracy of 96.27% for various fall directions.

However, the aforementioned methods lead to higher model complexity and greater computational burdens when improving recognition accuracy. Therefore, a critical challenge lies in the development of a lightweight model that can be seamlessly integrated into miniaturized devices.

Building on the previous discussion, this paper introduces a domain-adversarial adaptation approach that can be used to recognize driving behavior, named MCT-CNN-LSTM, which enhances the ability to extract multi-channel and multi-scale radar signal features. MCT-CNN-LSTM is composed of three key components. First, radar distance and Doppler information are extracted via Short-Time Fourier Transform (STFT). Next, dimensionality reduction is carried out using PCA, and a specially designed feature extractor captures relevant multi-scale information within the radar signals. Furthermore, Efficient Channel Attention (ECA) is integrated to sharpen the model’s emphasis on crucial data, aiding in the identification of essential features related to different driving behaviors. Finally, adversarial learning is employed to reduce domain mismatches between the source and target domains, which helps decrease data distribution variations across the domains, thereby improving the model’s capacity to identify driver behavior in real-world driving environments.

The contribution of this article is summarized as follows:

(1) A multi-scale parallel sub-network design is used to capture both short-range and long-range dependencies in radar signals, enabling the model to learn richer feature representations at multiple scales.
(2) The cross-space learning method is employed to fuse the outputs of parallel sub-networks, effectively capturing the pairwise relationships at the sample level of radar spectrograms following PCA dimensionality reduction. This approach enhances the representation of global contextual information within the spectrogram and improves the aggregation of relevant features.
(3) Combined with the LSTM and ECA mechanism, it significantly enhances the performance of driving behavior detection while requiring fewer parameters. This reduction in computational complexity offers an effective solution for deploying miniaturized devices within vehicles.
(4) This method is validated on real-world driving scenarios for the recognition of driver behavior, achieving a recognition accuracy of 97.3%. It can also be integrated into a miniaturized device, referred to as the MCCT Device.

**Figure 1:**
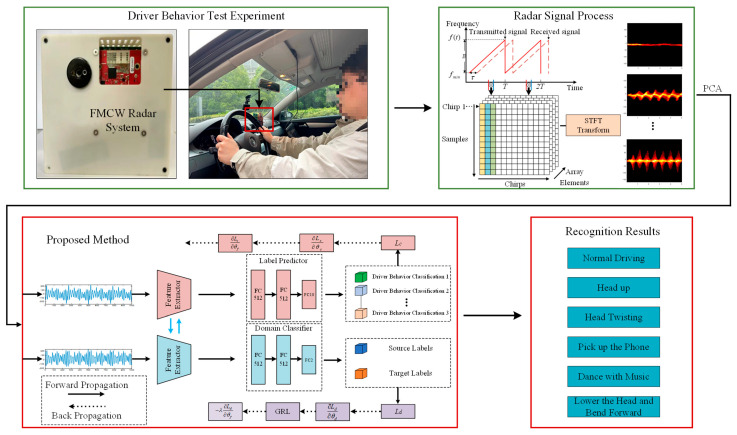
General framework of the article.

The framework of this paper is illustrated in Figure 1. Initially, we developed an FMCW radar system and conducted driving behavior experiments. Upon collecting the radar data, we processed it to generate radar micro-Doppler images. These images were then subjected to PCA-based dimensionality reduction before being fed into the proposed MCT-CNN-LSTM network for training. Finally, the model’s performance was evaluated and validated on six distinct types of driving behaviors.

The organization of the article is outlined as follows: Section 2 describes the measurement setup. The proposed method is introduced in Section 3. The experimental verification of the development method is presented in Section 4. Finally, Section 5 provides the conclusion and discusses future work.

## 2. Measurement Setup

### 2.1. Miniaturized FMCW Radar System

**Figure 2:**
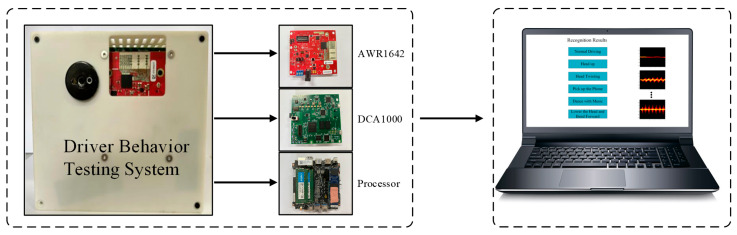
Configuration of the driving behavior testing system.

Figure 2 illustrates the system designed in this article, incorporating the AWR1642, DCA1000, an Intel Core i5-8279U processor, and a laptop for visualization. Notably, when the FMCW radar is deployed on this compact device, it occupies minimal space, allowing easy integration into vehicles and demonstrating its potential to meet the needs of resource-constrained embedded systems in the future.

### 2.2. Principle of FMCW Radar Transmission and Reception

FMCW radar systems transmit a chirping signal. The chirp’s frequency changes linearly over time, and a collection of chirps forms a frame that serves as an observation window for processing radar signals. Several factors related to the chirp, such as frequency slope and scanning band, influence the performance of the system.

**Figure 3:**
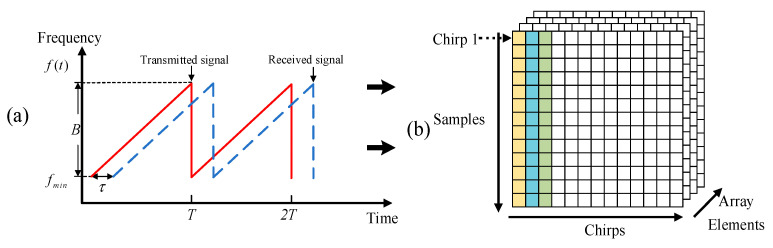
(**a**) The time–frequency diagram of the transmitted and received signals; (**b**) FMCW radar data block format.

As depicted in Figure 3a, the mixed output signal M(t) can be obtained by combining the transmitted signal with the received one in the FMCW radar sensor as follows [7]:

(1) $$M(t)=\exp\left(j\cdot 2\pi \cdot \left(f_{c}\tau +\frac{B}{T}t\tau -\frac{1}{2}\frac{B}{T}\tau^{2}\right)\right)$$

where B represents the bandwidth; j represents the imaginary unit; t is the time; $f_{c}$ denotes the frequency of carrier; T refers to the sweep time; $\tau =2\left(R+vt\right)/c$ is the round-trip delay; and c is the speed of light. This is calculated assuming there is a driver at a distance of R from the radar moving with a radial velocity of v.

The data collected through ADC sampling and transmitted by the DCA1000 cannot be immediately processed in its raw form (Bin files). Initially, the data must be rearranged into three-dimensional data blocks. As shown in Figure 3b, each column in this block represents all the samples from a single chirp signal. In each data frame, the columns correspond to distinct chirps, with the target data being sampled at multiple instances. The data collected from different array elements are structured into separate pages within the 3D block. Each page contains an intermediate frequency (IF) signal, which results from mixing and filtering operations. Following this, the data undergo a balancing procedure to ensure consistency and accuracy. Additionally, irrelevant signals are suppressed.

## 3. Proposed Method

To comprehensively extract driving behavior information from radar range-Doppler spectrograms, we propose an improved MCT-CNN-LSTM method. This algorithm integrates an MCT feature extraction module for a domain-adversarial neural network (DANN) to extract radar spectrogram features from multiple channels and angles. This design improves the ability of the model to capture temporal dependencies while emphasizing key features through the use of an attention mechanism.

### 3.1. MCT-CNN-LSTM Method

Subsubsection

**Figure 4:**
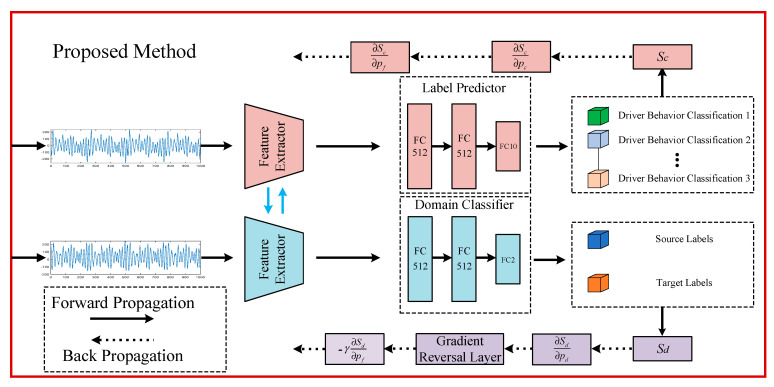
Flow diagram of the driver behavior recognition model.

The proposed MCT module is combined with the DANN to develop a driving behavior recognition model tailored to real-word driving conditions. A flowchart of the model is shown in Figure 4.

During the training of source domain data, action labels were employed to establish a supervised learning setup. In contrast, when sufficient labels are unavailable, the target domain data are classified using shared network parameters. 

**Figure 5:**
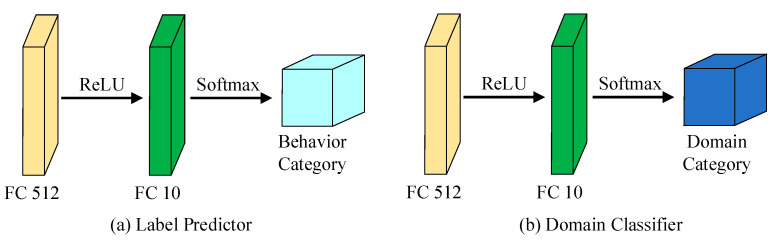
The composition of label predictor and domain classifier.

As shown in Figure 5, the label predictor and domain classifier both feature comparable fully connected layers, each followed by a softmax classifier. The key difference between the two is found in the final fully connected layer of the label predictor, where the number of neurons corresponds to the total categories involved in the classification task.

The primary function of the label predictor is to classify actions based on the feature information extracted by the feature extractor. As a critical component of the DANN, it is responsible for performing the driving behavior classification task. The label predictor is composed of several fully connected layers that process the feature vectors produced by the feature extractor, extracting more discriminative features. The final layer of the label predictor is a softmax classifier, which maps the processed feature vectors to specific fault categories.

Similarly, the domain classifier is responsible for determining whether the input features belong to the source domain or the target domain. This component plays a crucial role in the DANN, facilitating domain-adversarial learning. Like the label predictor, the domain classifier consists of several fully connected layers, which process the feature vectors output by the feature extractor to identify features that can effectively distinguish between different domains.

### 3.2. Feature Extraction Module

**Figure 6:**
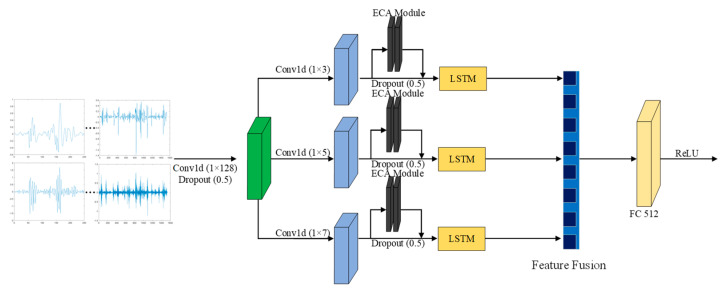
Feature extraction component.

The proposed feature extraction architecture is depicted in Figure 6. This module consists of several components: (1) Wide convolutional kernels are utilized to facilitate the initial extraction of driver behavior Doppler features within radar signals. (2) Multi-scale convolutions are implemented to capture features from various perspectives, thereby improving the model’s ability to process and interpret radar signals more effectively. (3) An LSTM is integrated to improve the network’s capability in extracting temporal information from the signal, capturing the dynamics over time. (4) The ECA is incorporated to emphasize the most significant features, optimizing the model’s performance by focusing on the most relevant information. (5) Dropout is employed to reduce overfitting and mitigate the time-consuming nature of training deep neural networks, improving generalization and efficiency.

The original radar signal was first transformed using the STFT to obtain the range-Doppler signal, which is then subjected to dimensionality reduction via PCA before being passed into the feature extraction component.

Suppose the spectrogram is a two-dimensional set of feature vectors X. The PCA transformation requires the transformation of X into a one-dimensional Y.

(2) Y=aX

The PCA method needs to find the maximum of the following equation to find the linear mapping a.

(3) $$\max\left(a^{T}cov(X)a\right)$$

Here, cov(X) is the covariance of the set of spectral map eigenvectors.

To enhance the process of extracting temporal features from the radar range-Doppler spectrogram, a 1-D convolution with wide convolutional kernels was initially applied in the shallow convolutional layer, followed by processing in the feature extraction component. The kernel dimensions varied across the branch structure, enabling the extraction of more detailed feature information from various perspectives. The features gathered from the different-scale layers were subsequently combined. To enhance generalization, dropout was applied, randomly deactivating specific neurons with a given probability. In the final stage, fully connected layers were incorporated to establish a unified feature extraction framework.

**Figure 7:**
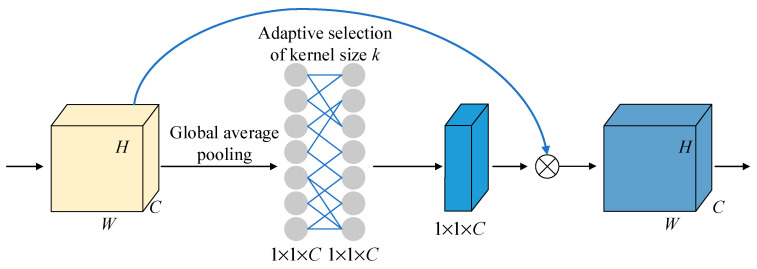
ECA-Net schematic diagram.

In the process of feature extraction, redundant information can significantly affect both the performance of the network and the effectiveness of feature selection. To address this challenge, the ECA [24] method focuses on prioritizing important information, thus improving both operational efficiency and generalization ability. Figure 7 illustrates the structure of the ECA module used in this paper. The ECA module is an efficient attention mechanism designed to enhance the model’s ability to focus on important information by increasing the weight of critical feature channels, thereby improving the efficiency and accuracy of feature extraction. Specifically, the ECA module compresses each channel of the feature map into a single feature value through global average pooling (GAP) and then applies an adaptive one-dimensional convolutional kernel to weigh these feature values. The size of the convolutional kernel is dynamically calculated based on the number of input feature channels, ensuring the efficient capture of inter-channel dependencies. Finally, Sigmoid is used to map the weighted feature values to a range of 0 to 1, which serves as attention weights that are multiplied with the original feature map channel-wise, thereby enhancing the expression ability of the critical feature channels. This design not only reduces the number of parameters but also improves the model’s sensitivity to important features by adaptively adjusting channel weights, providing more effective feature representations for driving behavior recognition. The proposed ECA approach is realized by incorporating a 1×1×C convolutional layer following the global average pooling layer, which replaces the traditional fully connected layer. This module effectively circumvents dimensionality reduction issues and yields an improved performance with fewer parameters.

### 3.3. Parameter Update of MCT-CNN-LSTM

In this study, the cross-entropy loss function was utilized for classification, and its mathematical formulation is provided below:

(4) $$S_{c}=\frac{1}{C}\sum_{i}S_{i}=-\frac{1}{C}\sum_{i}\sum_{c=1}^{T_{c}}x_{ic}\log(y_{ic})$$

(5) $$S_{d}=\frac{1}{C}\sum_{i}S_{i}=-\frac{1}{C}\sum_{i}\sum_{c=1}^{T_{d}}x_{ic}\log(y_{ic})$$

where C refers to the number of samples; $T_{c}$ and $T_{d}$ represent the count of classes; $S_{c}$ corresponds to the sample classification loss; and $S_{d}$ represents the loss for domain classification. The domain classifier considers both source and target domains, denoted as $T_{d}=2S_{d}$. For the label predictor, there are six driving behavior categories; thus, $T_{c}=6S_{c}$. $x_{ic}$ denotes the signum function; $x_{ic}=1$ when the true category of sample i matches class c; and $x_{ic}=0$ otherwise. $y_{ic}$ represents the predicted probability of the radar signal.

During the process of training the model, the objective is to minimize the classification error while maximizing the domain classification error. This dual objective facilitates the accurate classification of driving behavior. To accomplish this, the model is trained by minimizing the loss function, which comprises two main components: the loss $S_{c}$ within the actual and predicted sample labels and the loss $S_{d}$ between the actual and predicted domain labels. The specific computation formula is provided below:

(6) $$S(p_{f},p_{c},p_{d})=\sum_{i=1}^{C}S_{c}^{i}(p_{f},p_{c})-\gamma \sum_{i=1}^{C}S_{d}^{i}(p_{f},p_{d})$$

where γ represents the tunable parameter; $p_{f}$ denotes the parameters of the feature extractor; $p_{c}$ refers to the settings of the label estimator; and $p_{d}$ stands for the parameters of the domain identifier. For parameter updates, we employed the Adam optimization algorithm, as outlined below:

(7) $$\left({\hat{p}}_{f},{\hat{p}}_{c}\right)=\arg\underset{p_{f},p_{c}}{\min}S_{c}(p_{f},p_{c},{\hat{p}}_{d})$$

(8) $${\hat{p}}_{d}=\arg\underset{p_{d}}{\max}S_{d}({\hat{p}}_{f},{\hat{p}}_{c},p_{d})$$

where $p_{d}$ held constant, and the focus was on training $p_{f}$ and $p_{c}$ to reduce the label predictor loss $S_{c}$. Conversely, when $p_{f}$ and $p_{c}$ are fixed, $p_{d}$ is trained to minimize the loss $S_{d}$. This process ultimately aims to minimize the overall network loss S.

The inclusion of a gradient reversal layer (GRL) within the DANN leads to an automatic reversal of the gradient direction during backpropagation, while a constant transformation is applied during forward propagation. Moreover, in the DANN framework, the learning rate is adaptively modified at each iteration.

(9) $$\nu_{p}=\frac{\nu_{0}}{1+{\left(\omega \cdot \theta \right)}^{\sigma }}$$

where $\nu_{0}=0.01$ represents the starting learning rate, and θ is the relative measure in each iteration, determined by the ratio of the current iteration number to the total number of iterations. Additionally, ω=10 and σ=0.75 are optimization parameters.

## 4. Experiments

This section presents the experimental validation of MCT-CNN-LSTM by recognizing driving behaviors within an open campus area. The method is comprehensively compared with several effective human activity recognition techniques. The subsequent sections are structured as follows. First, the driving behavior data collection experiment is presented, along with the definition of various driving behaviors. Next, the training processes and comparison methods are introduced. Lastly, a comprehensive analysis of the experimental results is provided.

### 4.1. Experimental Details

**Figure 8:**
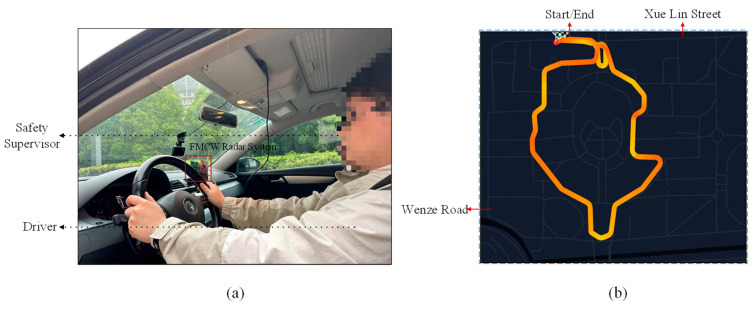
Driving data collection experiment (**a**) Driving internal monitoring environment, (**b**) vehicle route.

The measurement system relied on AWR 1642, DCA1000, and a processor, as demonstrated in Figure 2. Figure 8a illustrates the vehicle’s interior, where the driver performs the necessary driving behaviors during the experiment while a safety officer is seated in the front passenger seat to ensure safety. As depicted in Figure 8b, the vehicle followed the specified lane on the campus route, with the vehicle speed not exceeding 30 mph.

### 4.2. Datasets

**Figure 9:**
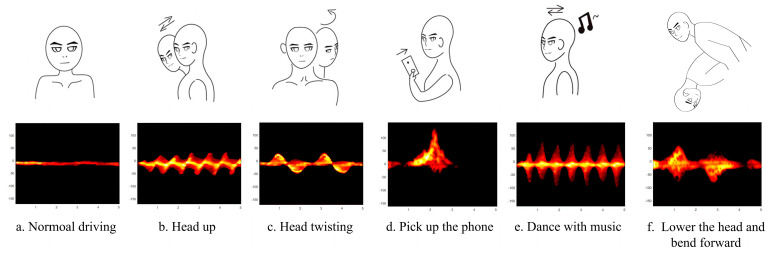
Radar range-Doppler map of driving behavior.

The data collection process lasted around one month and was conducted to ensure that the gathered data were sufficiently random. As outlined in Table 1, six volunteers (four males and two females) took part in the experiment, performing six different movements under real driving conditions: normal driving (ND), head-up (HU), head-turning (HT), picking up the phone (LP), dancing to music (DM), and lowering the head while bending forward (BD). Table 1 presents the testers’ information along with the number of actions performed during each test. It includes details such as the testers’ gender, age, and the number of ND, HU, HT, LP, DM, and BD actions collected for each tester. These activities were real-life actions, not simulated, and covered various directions and locations to promote dataset diversity. A total of 1000 samples were collected for each activity to maintain a dataset balance. The six driving behaviors are represented as radar range-Doppler maps in Figure 9.

### 4.3. Details of Training and Testing

In total, 80% of the final dataset was randomly selected for the training set, while the remaining 20% served as the test set. Training and testing were performed on an NVIDIA A100 Tensor Core GPU, utilizing Python 3.7 and PyTorch 1.10.

### 4.4. Experimental Results

**Figure 10:**
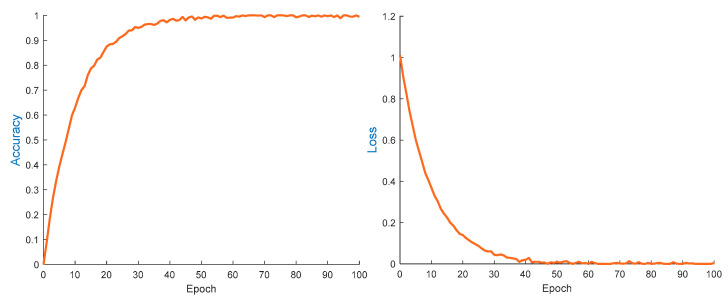
Results of accuracy and loss function of the MAC-CNN-LSTM method.

As illustrated in Figure 10, while training on the cross-validation set, the driver behavior recognition accuracy of the MCT-CNN-LSTM method approaches nearly 100%. The accuracy increases rapidly between 0 and 20 epochs, then converges to its final value at around 50 iterations, after which it stabilizes. Similarly, the loss function curve of the MCT-CNN-LSTM method demonstrates rapid convergence at around 20 iterations, followed by a gradual leveling off. The loss value approaches zero and exhibits minor fluctuations thereafter.

**Figure 11:**
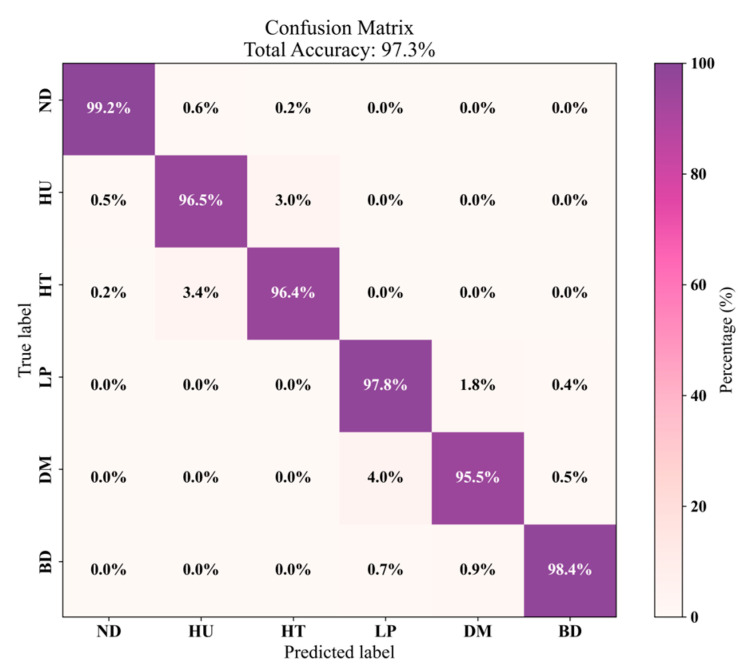
MCT-CNN-LSTM classification matrix.

Figure 11 illustrates the confusion matrix outcomes for the MCT-CNN-LSTM classification. The primary sources of errors, as shown in the figure, involve the misclassification of ND, HU, and HT, as well as LP, DM, and BD. These errors can be mainly attributed to the resemblance in micro-Doppler features observed in the early stages of the “pick up the phone” and “lower the head and bend forward” activities. In particular, when the “lower the head and bend forward” movement has a smaller amplitude, it is frequently misidentified as “dance with music”. Additionally, the confusion between HU, HT, and ND occurs because of the reduced amplitude in the “head up” and “head twisting” movements. When the amplitude is minimal, the FMCW radar struggles to capture the motion accurately, resulting in a very slight Doppler shift that the system recognizes as no movement, leading to misclassification. In future work, we intend to perform a more detailed analysis of the micro-Doppler features to identify finer characteristics that could facilitate the differentiation of small-amplitude movements, such as “lower the head and bend forward”. Furthermore, we aim to optimize the model’s architecture to enhance its ability to capture the subtle variations inherent in low-amplitude movements.

**Figure 12:**
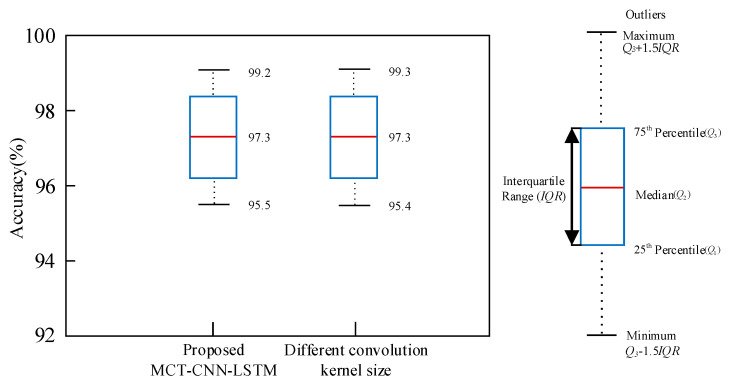
Comparison of recognition accuracy between the proposed method with different convolution kernel sizes.

The influence of convolution kernel size and the number of LSTM layers on the results is discussed through ablation experiments. Initially, different kernel sizes, including 1 × 3, 1 × 5, 1 × 7, and 1 × 9, are tested. From these four kernel sets, three combinations (1 × 3, 1 × 5, and 1 × 7) are selected for comparison with the MCT-CNN-LSTM method. As shown in Figure 12, “Different convolution kernel size” represents the average recognition value of any of the three combinations of convolution kernels mentioned above. The results indicate that the combination of different convolution kernels has a minimal impact on the final driving behavior recognition outcomes. This can be attributed to the implementation of multi-scale convolution, which extracts feature information from multiple perspectives, thereby improving the classification accuracy of driving behaviors. The primary objective of multi-scale convolution is to capture features at various scales using kernels of different sizes, enhancing the model’s capacity to comprehend the input data.

**Figure 13:**
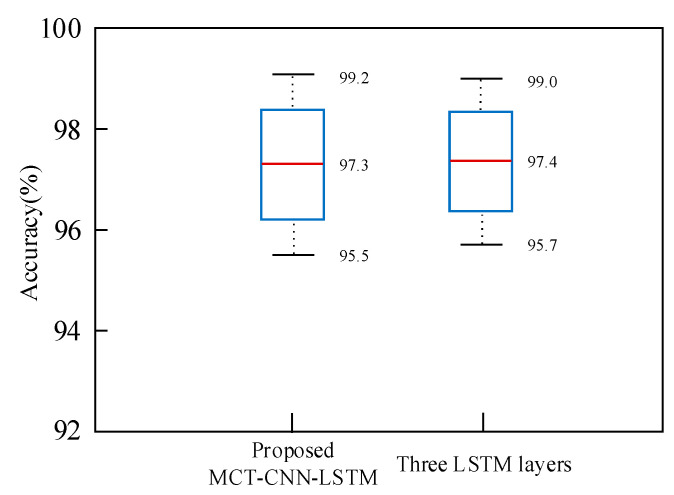
Comparison of recognition accuracy between the proposed method with different LSTM layers.

Moreover, we conducted experiments with different numbers of LSTM layers (e.g., 2, 3, 4, and 5 layers) to investigate their impact on the model’s ability to capture temporal dependencies. While deeper LSTM layers have the potential to enhance the model’s performance on sequences with more complex temporal patterns, they may also lead to overfitting or increased computational burden. As illustrated in Figure 13, the average recognition performance with varying numbers of LSTM layers showed only slight, marginal improvements (1%) over the proposed MCT-CNN-LSTM model. This suggests that the MCT-CNN-LSTM model is not particularly sensitive to these hyperparameters.

Secondly, seven other state-of-the-art methods, including AlexNet [25], 1-D CNN [26], CNN-ECA [27], CNN-LSTM [28], CNN-LSTM-ECA, CNN-Channel Attention, and RFDANet [7], were chosen for comparison in the driver behavior recognition task. In Table 2, we can see that MCT-CNN-LSTM achieved the highest accuracy in recognizing ND, HT, LP, DM, and BD. The average classification accuracy increased from 78.2% to 97.3%, surpassing other methods by a minimum of 3.0%.

In our analysis of the complexity of the proposed MCT-CNN-LSTM method compared to other methods for identifying six types of driving behaviors, we observed that MCT-CNN-LSTM excels in efficiency. As shown in Table 3, it requires the fewest parameters at 5.1 million, outperforming methods like AlexNet with 18.3 million. Regarding FLOPs, MCT-CNN-LSTM utilizes 0.6 billion, which, while not the lowest, remains competitive. Its model size is 28.3 MB, indicating a modest reduction. Crucially, MCT-CNN-LSTM achieves a superior processing speed at 23.1 ms per inference and an acc./speed ratio of 4.2, demonstrating a balance of high recognition accuracy and faster processing compared to other methods, such as RFDANet with a 31.2 ms speed and an acc./speed of 3.0.

**Figure 14:**
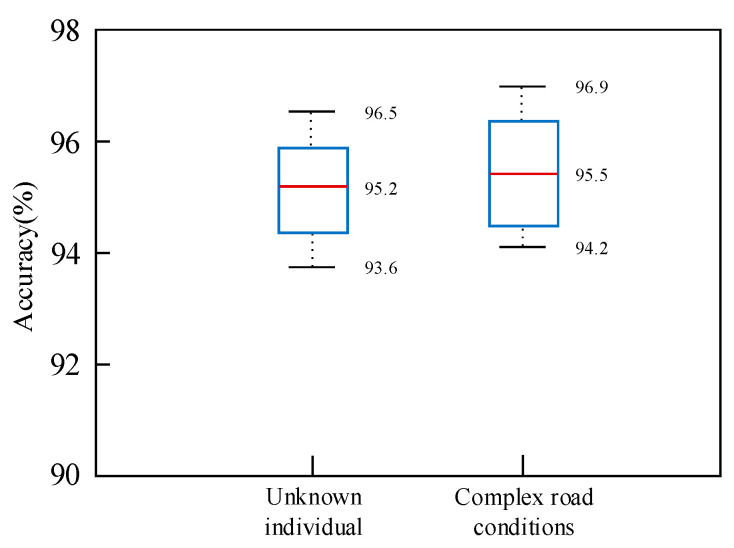
Results of the proposed method in recognizing unknown individual and complex road conditions.

Furthermore, we tested the model’s performance in recognizing unknown individuals and operating in complex road conditions. As illustrated in Figure 14, the model achieves an average recognition accuracy of 95.2% for recognizing unknown individuals and 95.5% for operating in complex road conditions. These results correspond to reductions in accuracy of 2.1% and 1.8%, respectively, highlighting the model’s high robustness.

Ultimately, the proposed MCT-CNN-LSTM method is embedded into the miniaturized device (MCCT Device) illustrated in Figure 1. Three participants performed 100 trials for each action. For the test results presented in Table 4, TND is the count of correct recognitions for ND; THU is the count of correct recognitions for HU; THT is the count of correct recognitions for HT; TLP is the count of correct recognitions for LP; TDM is the count of correct recognitions for DM; and TBD is the count of correct recognitions for BD. Notably, misjudgments are observed only in the HT and DM actions, with 1–2 and 1–3 instances of misclassification, respectively. These findings demonstrate that the proposed method exhibits a high level of robustness.

## 5. Conclusions

This paper introduces a neural network model, MCT-CNN-LSTM, for classifying and recognizing multi-class driving behaviors based on FMCW radar signals. The multi-scale module of the model effectively extracts and filters features, capturing crucial information related to Doppler signal variations and subsequently reconstructs the feature extractor within the conventional DANN framework. The MCT-CNN-LSTM model effectively extracts common features from dual-domain data, thereby addressing the issue of inadequate shared feature extraction between the dual domains. This approach’s effectiveness was confirmed through experimental tests using real measured datasets. Ultimately, the proposed system was integrated into a compact device, and a driving behavior recognition experiment was performed, highlighting the robustness of our method. The MCT-CNN-LSTM model offers an innovative solution for driving behavior recognition, with future research aiming to broaden the dataset to cover more complex and diverse traffic scenarios.

## Figures and Tables

**Table 1:** The information of driver testers.

ID	Gender	Age	ND	HU	HT	LP	DM	BD
1	Male	26	199	195	195	195	195	200
2	Male	46	99	100	105	105	100	100
3	Male	29	102	105	100	100	105	100
4	Female	32	201	200	140	160	200	180
5	Male	22	199	200	220	230	200	210
6	Female	23	200	200	240	210	200	210

**Table 2:** Recognition performance of the proposed method with different classifier models.

Models	(a) ND	(b) HU	(c) HT	(d) LP	(e) DM	(f) BD	Avg
AlexNet	84.5%	79.5%	80.2%	80.7%	71.4%	72.6%	78.2%
1-D CNN	97.0%	82.4%	81.7%	83.7%	73.2%	76.1%	82.4%
DANN	97.5%	83.1%	81.9%	84.2%	78.6%	80.3%	84.3%
CNN-ECA	97.3%	81.6%	82.4%	84.5%	79.8%	78.2%	84.0%
CNN-LSTM	98.0%	84.4%	82.6%	84.2%	82.9%	82.5%	85.8%
CNN-LSTM-ECA	98.4%	96.6%*	96.2%	86.5%	84.5%	85.1%	91.2%
CNN-Channel Attention	99.0%	85.2%	87.1%	90.4%	88.3%	89.6%	89.9%
RFDANet	99.0%	92.8%	92.6%	97.4%	90.8%	93.2%	94.3%
MCT-CNN-LSTM	99.2% *	96.5%	96.4% *	97.8% *	95.5% *	98.4% *	97.3% *

* Means the best result after comparison.

**Table 3:** Parameters, FLOPs, size, speed, and acc./speed comparison results.

Models	Params (M)	FLOPs (G)	Size (MB)	Speed (ms)	Acc./Speed
AlexNet	18.3	1.5	58.4	42	1.9
1-D CNN	5.5	0.4 *	8.9*	20.5 *	4.0
DANN	3.4	0.6	5.6	25.9	3.3
CNN-ECA	5.8	0.5	9.4	21.5	3.9
CNN-LSTM	6.5	0.4 *	26.2	24.4	3.5
CNN-LSTM-ECA	10.3	0.5	25.9	26.7	3.4
CNN-Channel Attention	7.8	0.7	25.3	25.6	3.5
RFDANet	8.3	1.3	21.1	31.2	3.0
MCT-CNN-LSTM	5.1 *	0.6	28.3	23.1	4.2 *

* Means the best result after comparison.

**Table 4:** The test result of the MCT Device for six driving behaviors.

ID	Age	TND	THU	THT	TLP	TDM	TBD
Tester-1	22	100	100	100	100	99	100
Tester-2	41	100	100	98	100	97	100
Tester-3	27	100	100	99	100	98	100

## Data Availability

The dataset is available on request from the authors.
